# *Hedysarum langranii* *sp. nov.* (Fabaceae, Hedysareae), a New Species From China, and Supplementary Descriptions of *H. smithianum* and *H. dentatoalatum*

**DOI:** 10.1002/ece3.72950

**Published:** 2026-01-16

**Authors:** Pei‐Liang Liu, Yong‐Xing Qin, Lu‐Lu Xun, Bin Li, Yuan Lu, Ming Yue

**Affiliations:** ^1^ Key Laboratory of Resource Biology and Biotechnology in Western China, Ministry of Education Northwest University Xi'an Shaanxi China; ^2^ Shaanxi Engineering Research Centre for Conservation and Utilization of Botanical Resources Xi'an Botanical Garden of Shaanxi Province (Institute of Botany of Shaanxi Province) Xi'an Shaanxi China

**Keywords:** Fabaceae, Leguminosae, new species, phylogenetic incongruence, phylogeny, taxonomy

## Abstract

*Hedysarum langranii* sp. nov. (Fabaceae, Hedysareae) is described and illustrated from Shaanxi, Henan and Gansu, China. This new species is morphologically similar to *H. smithianum*, but can be distinguished by its glabrous stipules (vs. sparsely pubescent), standard petal 9–11 mm long (vs. 12–13 mm long), keel petals 12–13 mm long (vs. 14–15 mm long), androecium 13–15 mm long (vs. 16–17 mm long), loment articles 4–5 mm wide (vs. 5–7 mm wide). The new species can be easily distinguished from *H. dentatoalatum* by its papyraceous leaflets (vs. membranaceous), corolla pale purple or pink (vs. pale yellow); loment articles 5–7 × 4–5 mm, without prickle on the veins, with minute irregular teeth along both sutures (vs. 8–12 × 10–12 mm, with prickles on the veins, with wings and prominent teeth along both sutures). The phylogenetic tree based on the nuclear sequences shows that *H. langranii* is sister to *H. dentatoalatum*, while the tree based on the plastid sequences shows that *H. langranii* is sister to a clade consisting of *H. smithianum* and *H. dentatoalatum.* The new species and *H. dentatoalatum* are both diploids with a chromosome number 2*n* = 14. Supplementary descriptions of *H. smithianum* and *H. dentatoalatum* are also provided. The incongruent phylogenetic position of *H. dentatoalatum* could be explained by an ancient hybridization hypothesis, but requires further investigation to determine the reason for this incongruence.

## Graphical abstract

*Hedysarum langranii* sp. nov. (Fabaceae, Hedysareae) is described from China based on morphological and phylogenetic evidence. Supplementary morphological descriptions of *H. smithianum* and *H. dentatoalatum* are provided.

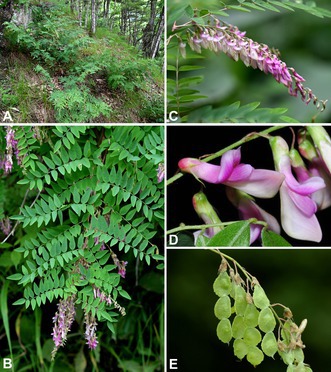

## 1 Introduction

*Hedysarum* L. (Fabaceae, Hedysareae) is a genus consisting of more than 160 species (Xu and Choi 2010). This genus has a temperate Northern Hemisphere distribution centered in Asia (Lock 2005; Xu and Choi 2010). Three sections of *Hedysarum* have been recognized mainly based on phylogenetic analyses, namely, *H*. sect. *Hedysarum*, *H*. sect. *Stracheya* (Benth.) B.H.Choi & H.Ohashi, and *H*. sect. *Multicaulia* (Boiss.) B.Fedtsch. (Duan et al. 2015; Liu, Wen, et al. 2017; Liu et al. 2019; Nafisi et al. 2019; Juramurodov et al. 2023). Morphology also supports the delimitation of the three sections, for example, *H*. sect. *Hedysarum* is characterized by its bright‐green leaves, visible lateral veins in the leaflets, compressed and unarmed loments, while *H*. sect. *Multicaulia* is characterized by its grayish‐green leaves, obscure lateral veins in the leaflets, and biconvex loments which always possess prickles, bristles, and ribs (Duan et al. 2015; Liu, Wen, et al. 2017). *Hedysarum* sect. *Stracheya* is sister to *H*. sect. *Hedysarum* (Liu, Wen, et al. 2017), and its strongly reduced stem is likely to be an adaptation to the psychric habitat in the Tibetan Plateau and the Himalayas (Duan et al. 2015; Liu, Wen, et al. 2017).

New species of *Hedysarum* have been described in Central and Western Asia in recent years (e.g., Juramurodov et al. 2021; Chaghamirzaei et al. 2022; Kandemi̇r et al. 2023; Ertuğrul et al. 2024, 2025; Oğur 2025). In China, 41 species of *Hedysarum* were recognized in *Flora of China* (Xu and Choi 2010). After the publication of *Flora of China*, five new species have been described (i.e., Choi et al. 2011; Zhao 2012; Liu, Wei, et al. 2017; Liu et al. 2019, 2024). To date, 46 species of *Hedysarum* have been found in China. In this paper, a new species of *Hedysarum* is described from China based on morphological and phylogenetic evidence. This paper contributes to our knowledge of species diversity of the ecologically and economically important plant Family Fabaceae, and updates the flora of China.

## 2 Material and Methods

### 2.1 Morphological Study

Specimens of the new and similar species were studied in herbaria HEAC, HIB, HITBC, HNWP, KUN, MO, NWTC, PE, WNU, WUK, and XBGH. Digital images of specimens in BJFC, GB, S, TIE, and UPS were studied through the Chinese Virtual Herbarium (www.cvh.ac.cn) and Sweden's Virtual Herbarium (herbarium.emg.umu.se). Herbarium code follows Index Herbariorum (sweetgum.nybg.org/science/ih). Field observations were conducted in Henan, Shaanxi and Shanxi Provinces, China.

### 2.2 Phylogenetic Reconstruction

#### 2.2.1 Taxon Sampling

A total of eight individuals from seven populations of the new species were sampled to test its phylogenetic position. Three individuals from two populations of *H. dentatoalatum* K.T.Fu and two individuals from two populations of *H. smithianum* Hand.‐Mazz. were sampled because of their morphological similarity with the new species. According to previous studies (Duan et al. 2015; Liu, Wen, et al. 2017; Liu, Wei, et al. 2017; Liu et al. 2019, 2024), additional species in *H*. sect. *Hedysarum* were included to construct a phylogenetic framework, and species in *H*. sect. *Stracheya* were selected as outgroups. Voucher information is available in Appendix 1.

#### 2.2.2 DNA Extraction, PCR and Sequencing

Genomic DNA was extracted using the Qiagen DNeasy Plant Mini Kit (Hilden, Germany) from silica‐gel dried leaf material. Polymerase chain reactions (PCR) were performed to amplify the nuclear ribosomal external transcribed spacer (ETS) and internal transcribed spacer (ITS), and the plastid *psb*A*‐trn*H, *trn*C*‐pet*N, *trn*L‐F, *trn*S‐G, and *pet*N*‐psb*M sequences. Primers and PCR conditions followed Liu, Wen, et al. (2017). The primers were used to sequence the amplicons in both directions. All sequences were deposited in GenBank and the accession numbers are provided in Appendix 1.

#### 2.2.3 Phylogenetic Analysis

The newly generated sequences together with the previously published data (Duan et al. 2015; Liu, Wen, et al. 2017; Liu, Wei, et al. 2017; Liu et al. 2019, 2024; Nuzhdina et al. 2018; Yurkevich et al. 2021) were used in phylogenetic analysis. The MUSCLE (Edgar 2004) implemented in Geneious Prime 2025.0.2 (www.geneious.com) was used to conduct multiple sequence alignments. The best‐fit nucleotide substitution models and the partition schemes were determined in PartitionFinder v.1.1.1 (Lanfear et al. 2012). One partition of the combined nuclear ETS and ITS sequences was suggested along with the HKY + G model. One partition of the combined plastid *psb*A*‐trn*H, *trn*C*‐pet*N, *trn*L‐F, *trn*S‐G and *pet*N*‐psb*M sequences was suggested along with the GTR + I + G model. The nuclear and plastid tree showed phylogenetic incongruence; therefore, they were not combined. Bayesian inferences (BI) were conducted in MrBayes v.3.2.5 (Ronquist and Huelsenbeck 2003; Ronquist et al. 2012) for 10,000,000 generations, and trees were sampled every 1000 generations. The first 25% trees were discarded, and the remaining trees were used to build a 50% majority‐rule consensus tree and posterior probabilities (PP). We also conducted the maximum likelihood (ML) and maximum parsimony (MP) analyses by using RAxML v.8.2 (Stamatakis 2014) and PAUP* 4.0a169 (Swofford 2002), respectively. The ML and MP bootstrap analyses were performed with 1000 replicates. Bootstrap support percentages (BSML, BSMP) from the ML and MP analyses were labeled on the corresponding branches of the BI trees.

### 2.3 Chromosome Number Count

Seeds of the new species (voucher: *P.L.Liu 201*, see Section 5.2 for details) and *H. dentatoalatum* (voucher: *P.L.Liu 1107*, see Section 5.3.2 for details) were collected from the field for chromosome number counts. Seeds were germinated in wet filter paper at room temperature. When the roots grew to ca. 5 mm long, they were treated in 2 mmol·L^−1^ 8‐hydroxyquinoline solution at room temperature for 4 h (Coe 1954; Liu et al. 2024). They were fixed in Carnoy's solution (mixture of acetic acid and ethyl alcohol, 1:3 volume) at 4°C overnight. The root tips were digested with 1 mol·L^−1^ hydrochloric acid at 60°C for 3 min, cleaned with tap water, stained with carbol fuchsin and squashed on glass slides. Well‐spread mitotic metaphase chromosomes were examined and photoed with a 100 × oil lens on a Nikon Eclipse 55i microscope.

## 3 Results

### 3.1 Morphological Comparison

Morphological comparison of the new species with *H. smithianum* and *H. dentatoalatum* was provided in Table 1. The new species is most similar to *H. smithianum* in gross morphology, but it has glabrous stipules, shorter petals, and smaller loment articles. The new species is differentiated from *H. dentatoalatum* by its smaller papyraceous leaflets, pale purple or pink corolla, and smaller loment articles without prickle on the veins and with minute irregular teeth along both sutures. Detailed diagnostic characters and full descriptions of these three species can be found in Section 5.

**TABLE 1:** Morphological comparison of *Hedysarum langranii*, *H. smithianum*, and *H. dentatoalatum*.

	*H. langranii*	*H. smithianum*	*H. dentatoalatum*
Stipules	Glabrous	Sparsely pubescent	Pubescent
Leaflets	Papyraceous; 18–30 × 5–9 mm	Papyraceous; 14–25 × 6–8 mm	Membranaceous; 35–50 × 10–22 mm
Corolla	Pale purple or pink	Pink	Pale yellow
Standard petal	9–11 × 3.5–4.5 mm	12–13 × 4–5 mm	12–14 × 5–7 mm
Wing petals	9–11 × 1.5–1.8 mm	11–12 × 1.5–1.8 mm	13–15 × 2.0–2.5 mm
Keel petals	12–13 × 4–5 mm	14–15 × 5.0–5.5 mm	17–19 × 6–7 mm
Androecium	13–15 mm long	16–17 mm long	18–19 mm long
Style	10–12 mm long	12–13 mm long	14–15 mm long
Loment articles	5–7 × 4–5 mm; without prickle on the veins; with minute irregular teeth along both sutures	6–8 × 5–7 mm; without prickle on the veins; with minute irregular teeth along both sutures	8–12 × 10–12 mm; with prickles on the veins; with wings and prominent teeth along both sutures

### 3.2 Phylogenetic Analysis

#### 3.2.1 Nuclear Data

**FIGURE 1:**
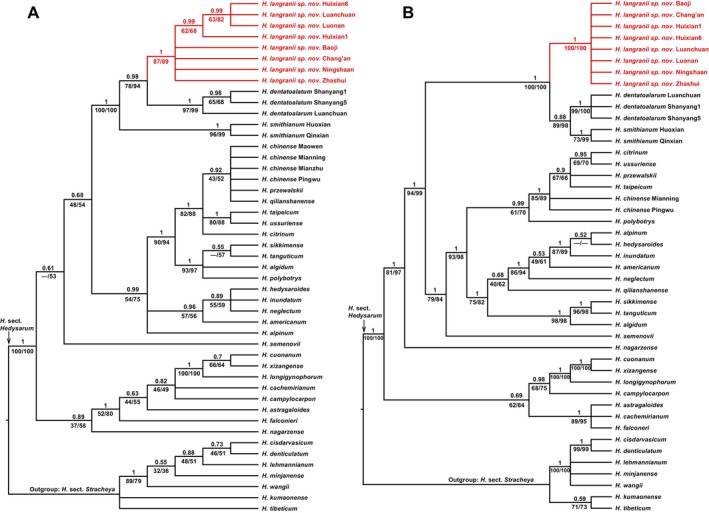
Bayesian 50% majority‐rule consensus trees based on the combined nuclear ETS and ITS sequences (A) and the combined plastid *psb*A*‐trn*H, *trn*C*‐pet*N, *trn*L‐F, *trn*S‐G and *pet*N*‐psb*M sequences (B). The Bayesian posterior probabilities (PP) are above the branches, and the maximum parsimony and the maximum likelihood bootstrap support percentages (BSMP, BSML) are below the branches. A dash (−) indicates a branch that is not found in the maximum parsimony or the maximum likelihood tree.

In the nuclear tree based on the combined ETS and ITS sequences (Figure 1A), all individuals of the new species formed a well‐supported clade (PP = 1, BSMP = 87%, BSML = 89%). The new species was sister to *H. dentatoalatum* (PP = 0.98, BSMP = 78%, BSML = 94%). *Hedysarum smithianum* was sister to the clade of the new species plus *H. dentatoalatum* (PP = 1, BSMP = 100%, BSML = 100%). The tree resolved the new species as a member of *H*. sect. *Hedysarum*.

#### 3.2.2 Plastid Data

In the combined plastid tree (Figure 1B), all individuals of the new species formed a well‐supported clade (PP = 1, BSMP = 100%, BSML = 100%). However, the plastid tree showed a different tree topology compared to the nuclear tree. The new species was sister to a clade comprised of *H. dentatoalatum* and *H. smithianum* (PP = 1, BSMP = 100%, BSML = 100%). *Hedysarum dentatoalatum* was sister to *H. smithianum* (PP = 0.88, BSMP = 89%, BSML = 98%).

### 3.3 Chromosome Number Count

**FIGURE 2:**
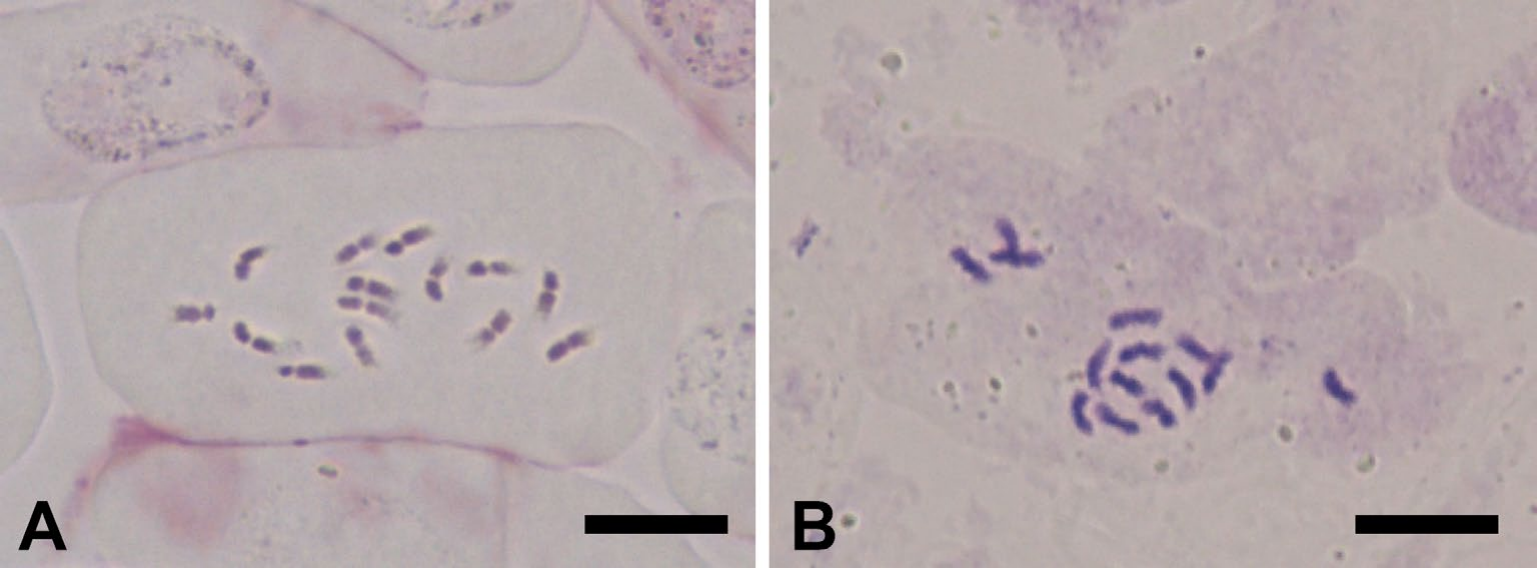
Mitotic metaphase chromosomes from root tips of *Hedysarum langranii* (A) and *H. dentatoalatum* (B). Bars = 10 μm.

For the new species, seven cells with well‐spread chromosomes from three different root tips were observed. All cells showed that the chromosome number of the new species was 2*n* = 14 (Figure 2A). For *H. dentatoalatum*, 15 cells with well‐spread chromosomes from seven different root tips were observed. All cells showed that the chromosome number of *H. dentatoalatum* was 2*n* = 14 (Figure 2B). Choi and Ohashi (2003) concluded that the basic chromosome number of *H*. sect. *Hedysarum* is *x* = 7. Therefore, *H. langranii* and *H. dentatoalatum* are both diploids.

## 4 Discussion

### 4.1 Morphological Differentiation

In gross morphology, *H. smithianum* and *H. langranii* are similar to each other, while *H. dentatoalatum* is substantially different from the former two species (Table 1). Both *H. smithianum* and *H. langranii* have smaller, papyraceous leaflets, pale purple or pink corolla, and smaller loment articles without prickles on the veins, with minute teeth along both sutures. In contrast, *H. dentatoalatum* has larger, membranaceous leaflets, pale yellow corolla, larger loment articles with prickles on the veins, with wings and prominent teeth along both sutures. The new species *H. langranii* can be distinguished from *H. smithianum* by its glabrous stipules, shorter petals, and smaller loment articles (Table 1).

Loment morphology is important in the taxonomy of *Hedysarum* (Xu and Choi 2010; Duan et al. 2015). *Hedysarum dentatoalatum* is peculiar in *H*. sect. *Hedysarum* by having prickles on the veins of loment articles because all other species in this section have plain loment (Fedtschenko 1948; Chrtková‐Žertová 1968; Isely 1998; Xu and Choi 2010). Besides *H. dentatoalatum*, the new species and *H. smithianum* are unique in *H*. sect. *Hedysarum* by having minute irregular teeth along both sutures of the loment because other species in this section have entire sutures (Fedtschenko 1948; Chrtková‐Žertová 1968; Isely 1998; Xu and Choi 2010). Additionally, corolla color, whether purple or yellow, serves as a practical and reliable character for distinguishing species in *Hedysarum* (Fedtschenko 1948; Chrtková‐Žertová 1968; Isely 1998; Xu and Choi 2010).

### 4.2 Distribution Pattern

**FIGURE 3:**
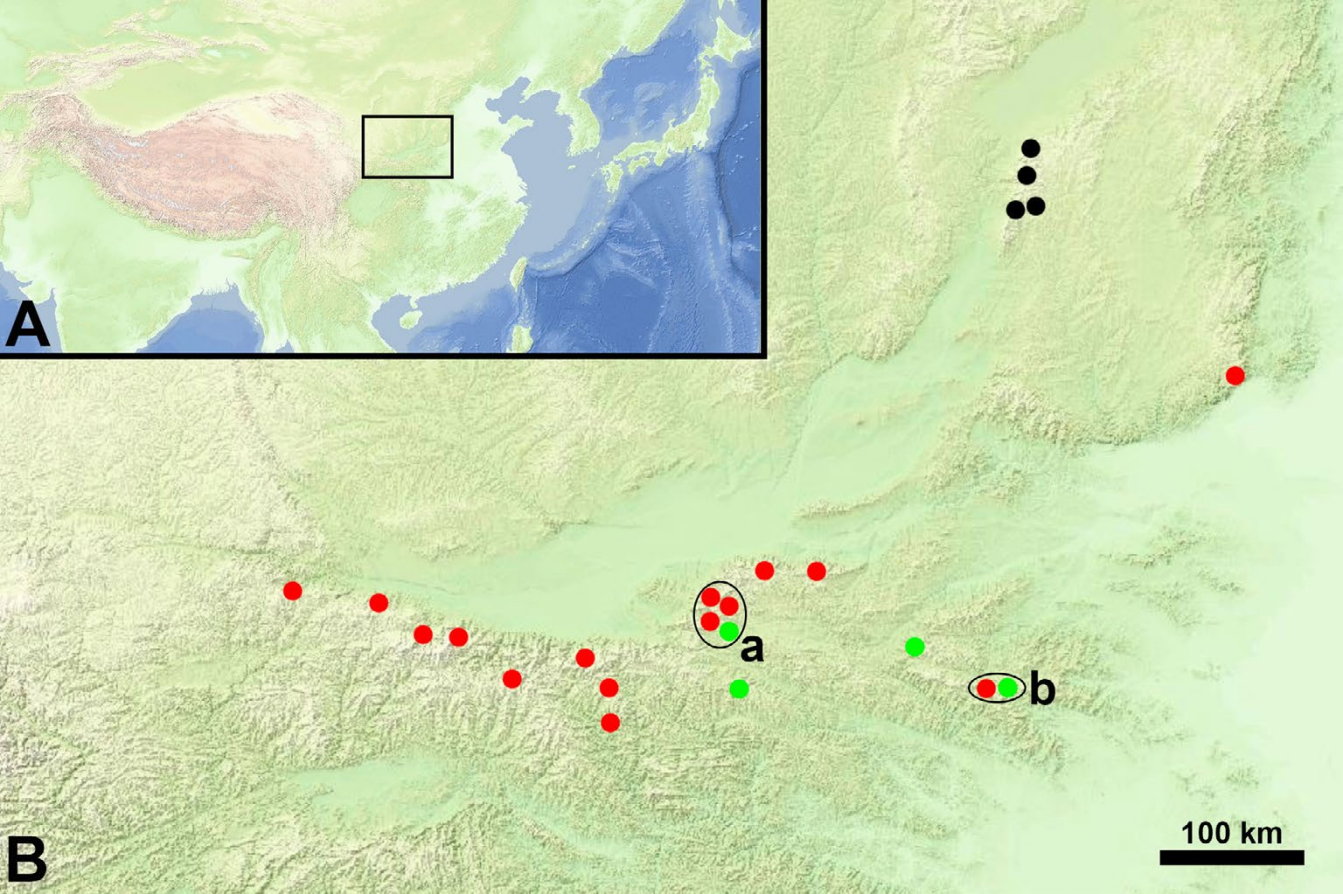
Distribution of *Hedysarum langranii* (red dots), *H. smithianum* (black dots) and *H. dentatoalatum* (green dots). The black frame in (A) is enlarged in (B). Black circles labeled a and b are explained in the discussion. Maps from map.tianditu.gov.cn.

In view of geographical distribution (Figure 3), *H. smithianum* is horizontally allopatric with *H. dentatoalatum* and *H. langranii*. This indicates that the divergence of *H. smithianum* can be attributed to geographical isolation. This pattern of allopatry has also been documented in *H. wangii* and its relatives (Liu et al. 2019) and in *H. qilianshanense* and its relatives (Liu et al. 2024). In contrast, the range of *H. dentatoalatum* falls within that of *H. langranii*. However, *H. dentatoalatum* and *H. langranii* inhabit different altitudinal zones. For example, in southeastern Shaanxi Province (shown by the black circle labeled a in Figure 3), *H. dentatoalatum* grows in 1200 m a.s.l. in Shangzhou, while *H. langranii* grows in ca. 1800 m a.s.l. in Linwei, Luonan and Lantian (see the specimens cited in Section 5). Similarly, in western Henan Province (shown by the black circle labeled b in Figure 3), *H. dentatoalatum* grows in 1500 m a.s.l. in Songxian, while *H. langranii* grows in 2100 m a.s.l. in Luanchuan (see the specimens cited in Section 5). In other words, *H. dentatoalatum* and *H. langranii* exhibit a distribution pattern of altitudinal segregation (Milla et al. 2008). To our knowledge, this represents the first documented case of altitudinal segregation between close relatives in *Hedysarum*. This pattern is distinctive for *Hedysarum*, as most of the congeners exhibit allopatric or sympatric distributions (Fedtschenko 1948; Chrtková‐Žertová 1968; Isely 1998; Xu and Choi 2010).

### 4.3 Flowering Time Divergence

The flowering time of *H. dentatoalatum* is May and June, while that of *H. smithianum* is July. This phenological divergence can promote the reproductive isolation between these two species. It has been well documented that flowering time divergence is an important isolating barrier (e.g., Savolainen et al. 2006; Pegoraro et al. 2016; Xu et al. 2020). The flowering time of *H. langranii* is June and July, which partly overlaps with those of *H. dentatoalatum* and *H. smithianum*. The role of flowering time differences in the speciation of *H. langranii* remains to be explored.

### 4.4 Cytonuclear Incongruence

Our phylogenetic trees showed obvious cytonuclear incongruence. *Hedysarum dentatoalatum* is sister to *H. langranii* in the nuclear tree (Figure 1A), while it is sister to *H. smithianum* in the plastid tree (Figure 1B). Many mechanisms can lead to conflicting gene trees, such as hybridization, incomplete lineage sorting, and others (Wendel and Doyle 1998; Steenwyk et al. 2023).

The incongruent position of *H. dentatoalatum* could be explained by a hypothesis that *H. dentatoalatum* originated from the hybridization between an ancestor of *H. smithianum* and an ancestor of *H. langranii*. As discussed above, *H. dentatoalatum* is substantially different from the other two species in morphology. Such a feature of hybridization offspring exhibit novel or extreme characters is known as transgressive segregation (Rieseberg et al. 1999), which is thought to be beneficial for the hybrids to use the niches that none of the parental species can utilize (Seehausen 2004; Peñalba et al. 2024).

Other mechanisms such as incomplete lineage sorting could also explain the incongruent position of *H. dentatoalatum*. Genome‐scale data and new methods of phylogenetic analysis can promote our understanding of phylogenetic incongruence (Som 2014; Fleming et al. 2023; Steenwyk et al. 2023; Shen et al. 2025).

## 5 Taxonomy

### 5.1 Key to *Hedysarum langranii*, *H. smithianum*, and *H. dentatoalatum*

1a. Leaflets membranaceous; corolla pale yellow; loment articles with prickles on the veins, with wings and prominent teeth along both sutures ……………………………………………… ***H. dentatoalatum***.

1b. Leaflets papyraceous; corolla pale purple or pink; loment articles without prickle on the veins, with minute irregular teeth along both sutures……………………………………………………………………………2.

2a. Stipules glabrous; standard petal 9–11 mm long; keel petals 12–13 mm long; androecium 13–15 mm long; loment articles 4–5 mm wide………………………………………………………… ***H. langranii***.

2b. Stipules sparsely pubescent; standard petal 12–13 mm long; keel petals 14–15 mm long; androecium 16–17 mm long; loment articles 5–7 mm wide……………………………………… ***H. smithianum***.

### 5.2 New Species

#### 5.2.1 *Hedysarum langranii* P.L.Liu, sp. nov. (*H.* sect. *Hedysarum*) (Figures 4 and 5)

**FIGURE 4:**
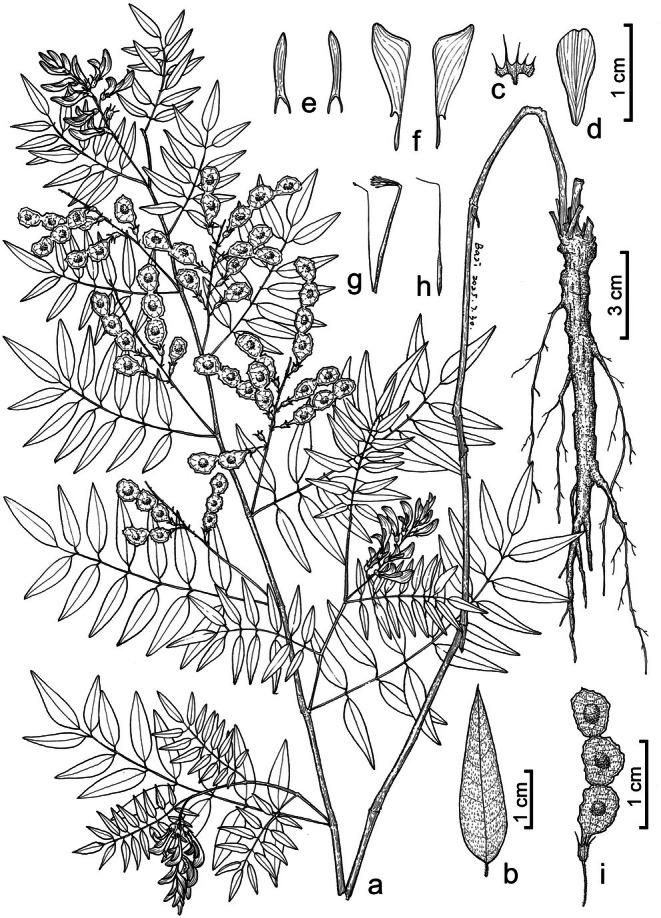
Illustration of *Hedysarum langranii*. (a) Flowering and fruiting plant; (b) leaflet, abaxial view; (c) calyx (split between an adaxial tooth and a lateral tooth); (d) standard petal; (e) wing petals; (f) keel petals; (g) androecium; (h) pistil; (i) legume. Drawn by Yi‐Fan Li.

**FIGURE 5:**
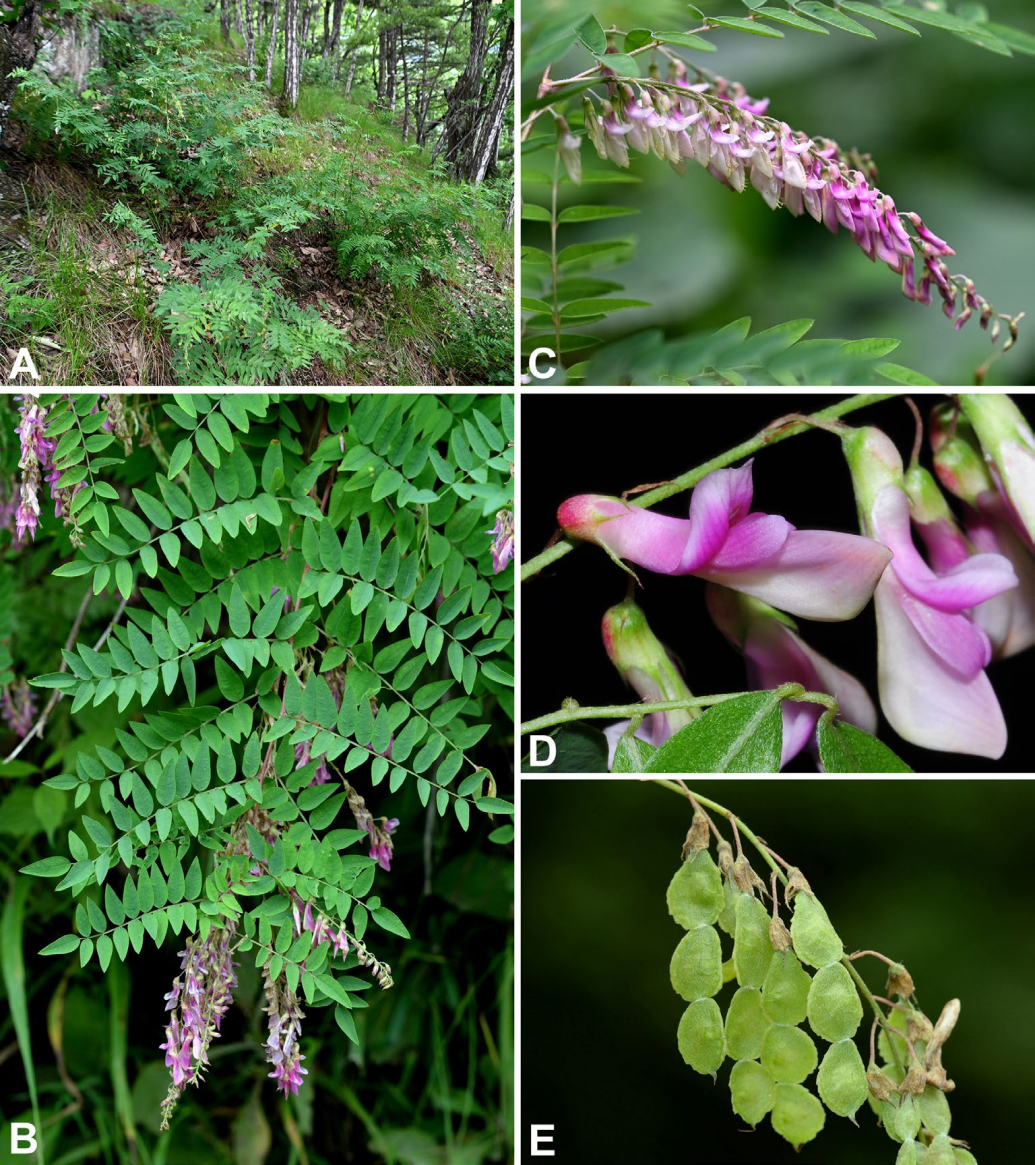
Photographs of *Hedysarum langranii* from the field. (A) Habitat; (B) upper part of a flowering plant; (C) raceme; (D) flowers; (E) legumes. (A and C) Photoed by Lu‐Lu Xun, (B, D and E) photoed by Pei‐Liang Liu.

**Type**. CHINA, Shaanxi Province, Ningshaan County, Caiziping, on mountain slope in shady forest, 1600 m above sea level (a.s.l.), 27 July 1990, *Z.H.Wu 90–749* (Holotype, WUK!, barcode WUK0476261; Isotype, WUK!, barcode WUK0476262).

**Diagnosis**. This new species is similar to *H. smithianum*, but can be distinguished by its glabrous stipules (vs. sparsely pubescent), standard petal 9–11 mm long (vs. 12–13 mm long), keel petals 12–13 mm long (vs. 14–15 mm long), androecium 13–15 mm long (vs. 16–17 mm long), loment articles 4–5 mm wide (vs. 5–7 mm wide). The new species can be easily distinguished from *H. dentatoalatum* by its papyraceous leaflets (vs. membranaceous), corolla pale purple or pink (vs. pale yellow), loment articles 5–7 × 4–5 mm, without prickle on the veins, with minute irregular teeth along both sutures (vs. 8–12 × 10–12 mm, with prickles on the veins, with wings and prominent teeth along both sutures) (Table 1).

**Description**. Perennial herbaceous plants, 0.6–0.9 m tall; main root 1–2 cm in diameter, woody. Stems cespitose, ascending, upper part branched; internodes sparsely pubescent. Leaves imparipinnate, alternate, 10–16 cm long; stipules connate, opposite to leaves, membranous, brown, glabrous, lower ones 10–17 mm long, becoming smaller in upper part of stem; rachises glabrous or sparsely pubescent; leaflets 11–21, opposite or alternate; petiolules 1–2 mm long, pubescent; leaflet blades lanceolate or ovate‐lanceolate, 18–30 × 5–9 mm, papyraceous, glabrous adaxially, sparsely pubescent abaxially, base broadly cuneate, apex acute and aristulate. Racemes axillary, including peduncles 5–10 cm long, with 15–25 flowers, peduncles sparsely pubescent; pedicels 3–4 mm long, sparsely pubescent; bracts linear, pubescent, 1–2 mm long; bracteoles 2, linear, 0.5–1.0 mm long; calyx tube 2.0–2.5 mm long, pubescent; calyx teeth 5, pubescent, linear‐triangular, the two adaxial teeth 1.5–2.0 mm long, the two lateral teeth 2.0–2.5 mm long, the abaxial tooth 2.5–3.5 mm long; corolla pale purple or pink; standard petal obovate, 9–11 × 3.5–4.5 mm, apex retuse, base attenuate; wing petals 9–11 × 1.5–1.8 mm, auricle linear, as long as claw, ca. 2 mm long; keel petals 12–13 × 4–5 mm, auricle triangular, ca. 0.5 mm long; androecium diadelphous, 13–15 mm long; ovary linear, glabrous, style 10–12 mm long. Legume a loment, compressed, divided into 2–4 articles; articles broadly ovate, 5–7 × 4–5 mm, pubescent, with reticulate veins, with minute irregular teeth along both sutures. Seeds brown‐yellow, ca. 3 × 2 mm.

**Phenology**. Flowering in June and July; fruiting in July and August; seeds mature in October.

**Distribution and Habitat**. *Hedysarum langranii* is distributed in southern Shaanxi, western Henan, and southeastern Gansu in China (Figure 3). It grows on stony slopes in temperate mixed coniferous‐broadleaf forests, 1500–2300 m a.s.l. The forests are dominated by *Quercus aliena* Blume var. *acuteserrata* Maxim. and *Pinus tabuliformis* Carrière, or by *Betula albosinensis* Burkill and *P. armandi* Franch.

**Etymology**. The epithet *langranii* was selected in honor of Prof. Lang‐Ran Xu (徐朗然, 1936–2020), who was a plant taxonomist and ecologist in Northwest A&F University. The Chinese vernacular name for this new species is 朗然岩黄耆 (lăng rán yán huáng qí).

**Other Specimens Examined (Paratypes)**. CHINA, Shaanxi Province, Zhen'an County, 10 June 1960, *Northwest University 273* (WUK!); Ningshaan County, 1800 m a.s.l., 12 July 1933, *H.W.Kung* 2999 (PE!, HIB!, WUK!); Ningshaan County, 33°43′41″ N, 108°22′49″ E, 2000–2100 m a.s.l., 30 July 2010, *Z.M.Jiang* et al. *1565* (WUK!); Huayin County, 2030 m a.s.l., 17 August 1966, *T.P.Wang 19734* (WUK!); Lantian County, 1800 m a.s.l., June 1960, *Z.X.Shen 217* (WUK!); Weinan (now Linwei District), 1800 m a.s.l., 7 July 1952, *T.P.Wang 15613* (WUK!, HIB!); Hu County (now Huyi District), 14 July 1951, *B.Z.Guo 353* (WUK!); Chang'an District, 1900 m a.s.l., 13 July 2016, *Z.Y.Chang* et al. *2016151* (WUK!); Chang'an District, 33°50′35″ N, 108°47′28″ E, 2299 m a.s.l., 9 July 2008, *S.F.Li* et al. *11004* (XBGH!); Zhashui County, 33°52′2″ N, 108°59′32″ E, 2183 m a.s.l., 5 October 2017, *P.L.Liu 201* (WNU! & WUK!); Zhashui County, 33°51′51″ N, 108°59′21″ E, 1988 m a.s.l., 19 July 2018, *P.L.Liu 415* (WNU! & WUK!); Luonan County, 34°16′15″ N, 109°49′18″ E, 1819 m a.s.l., 19 June 2024, *P.L.Liu 1647* (WNU! & WUK!); Weibin District, 34°14′54″ N, 107°10′32″ E, 1732 m a.s.l., 28 June 2024, *P.L.Liu 1649* (WNU! & WUK!); Mei County, 34°0′ N, 109°12′ E, 1800 m a.s.l., 22 June 2000, *G.H.Zhu* et al. *3053, 3055* (MO!); Taibai County, 33°54′ N, 107°42′ E, 1800 m a.s.l., 22 July 2000, *G.H.Zhu* et al. *3154* (MO!). Henan Province, Lingbao County, 5 August 2014, *J.M.Li* et al. *14080506* (HEAC!); Luanchuan County, 2100 m a.s.l., 23 August 2019, *P.L.Liu 576* (WNU! & WUK!); Luanchuan County, 25 July 2006, *C.S.Zhu 2006127* (HITBC!); Huixian County, 35°37′22″ N, 113°37′12″ E, 1540 m a.s.l., 10 July 2021, *P.L.Liu 1157* (WNU! & WUK!); Huixian County, 1500 m a.s.l., 15 July 1959, *Xinxiang Normal College 4153* (PE!); Huixian County, 1500 m a.s.l., 17 September 1959, *Xinxiang Normal College 4177* (PE!). Gansu Province, Tianshui, (Maiji District), 3 July 1985, *M.S.Yan 01747* (NWTC!).

### 5.3 Supplementary Descriptions

The new species is similar to *H. smithianum* and *H. dentatoalatum* in morphology, but the latter two species lacked detailed descriptions of flowers when they were published. Supplementary descriptions of these two species were provided based on more available specimens and field observations.

#### 5.3.1 *Hedysarum smithianum* Hand.‐Mazz., Symb. Sin. Pt. vii. 561. 1933 (Figure 6)

**FIGURE 6:**
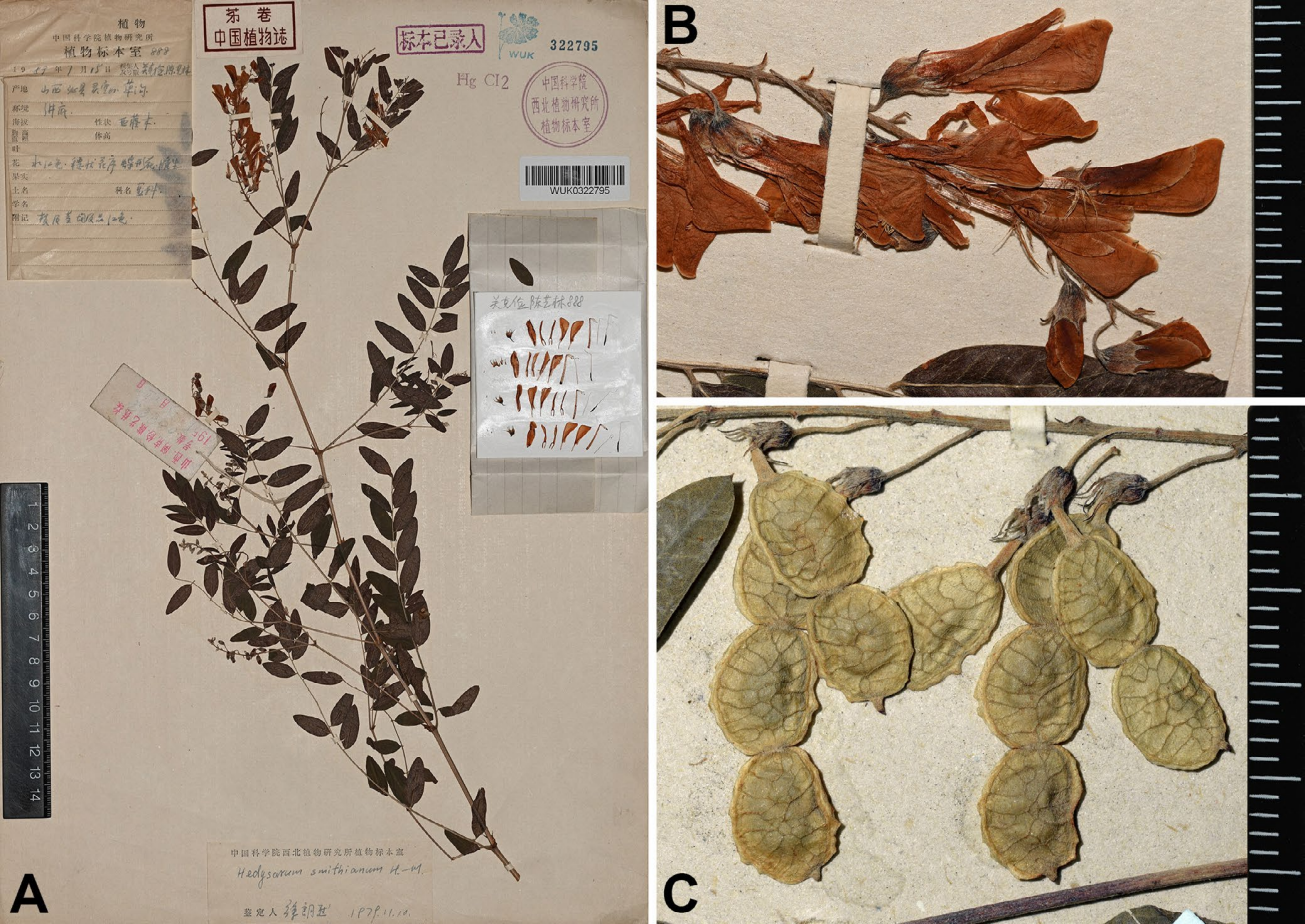
Specimens of *Hedysarum smithianum*. (A) Upper part of a flowering plant; (B) flowers; (C) legumes. (A and B) from *K.J.Guan & Y.L.Chen 888*; (C) from *K.J.Guan & Y.L.Chen 1096*. See the cited specimens for detailed information.

**Description**. Perennial herbaceous plants, ca. 0.6 m tall. Stems erect or ascending, upper part branched; internodes sparsely pubescent or glabrous. Leaves imparipinnate, alternate, 8–15 cm long; stipules connate, opposite to leaves, membranous, brown, sparsely pubescent, ca. 8 mm long in the middle of stem, becoming smaller in upper part of stem; rachises sparsely pubescent; leaflets 9–21, opposite or alternate; petiolules ca. 1 mm long, pubescent; leaflet blades ovate‐lanceolate, ovate or elliptical, 14–25 × 6–8 mm, papyraceous, glabrous adaxially, sparsely pubescent abaxially, base broadly cuneate, apex acute or obtuse, aristulate. Racemes axillary, including peduncles 5–8 cm long, with 15–20 flowers, peduncles sparsely pubescent; pedicels 4–5 mm long, sparsely pubescent; bracts linear, pubescent, 2.0–2.5 mm long; bracteoles 2, linear, 1.0–1.5 mm long; calyx tube 2.0–2.5 mm long, pubescent; calyx teeth 5, pubescent, linear‐triangular, the two adaxial teeth and the two lateral teeth 1.5–2.0 mm long, the abaxial tooth 2–3 mm long; corolla pink; standard petal obovate, 12–13 × 4–5 mm, apex retuse, base attenuate; wing petals 11–12 × 1.5–1.8 mm, auricle linear, as long as claw, ca. 2.5 mm long; keel petals 14–15 × 5.0–5.5 mm, auricle triangular, ca. 0.5 mm long; androecium diadelphous, 16–17 mm long; ovary linear, glabrous, style 12–13 mm long. Legume a loment, divided into 2–5 articles; articles broadly ovate, compressed, 6–8 × 5–7 mm, pubescent, with reticulate veins, with minute irregular teeth along both sutures.

**Phenology**. Flowering in July; fruiting in August to September; seeds mature in October.

**Distribution and Habitat**. *Hedysarum smithianum* is distributed in central Shanxi in China (Figure 3). It grows in subalpine meadow or temperate mixed coniferous‐broadleaf forest (dominated by *P. tabuliformis* and *Q. wutaishanica* Mayr), 1600–2300 m a.s.l.

**Specimens Examined**. CHINA, Shanxi Province, Chieh‐hsiuh Distr. (now Jiexiu City), Mien‐shan‐ye (Mt. Mianshan), in prato alpino, ca. 2300 m a.s.l., 3 October 1924, *H.Smith 7853* (Syntypes, GB!; S!; TIE!; UPS); Qinxian County (now Qinyuan County), Mt. Lingkongshan, Caogou, 15 July 1959, *K.J.Guan & Y.L.Chen 888* (HNWP!; WUK!); same locality, 24 August 1959, *K.J.Guan & Y.L.Chen 1096* (HNWP!; PE!, WUK!); Qinyuan County, Beilaigou, 2000 m a.s.l., 5 September 1991, *D.Z.Lu 91908* (BJFC!); Huoxian County (now Huozhou City), Licao, October 1972, *T.W.Liu s.n*. (PE00867707!).

#### 5.3.2 *Hedysarum dentatoalatum* K.T.Fu, Fl. Tsinling. 1(3): 448. 1981 (Figure 7)

**FIGURE 7:**
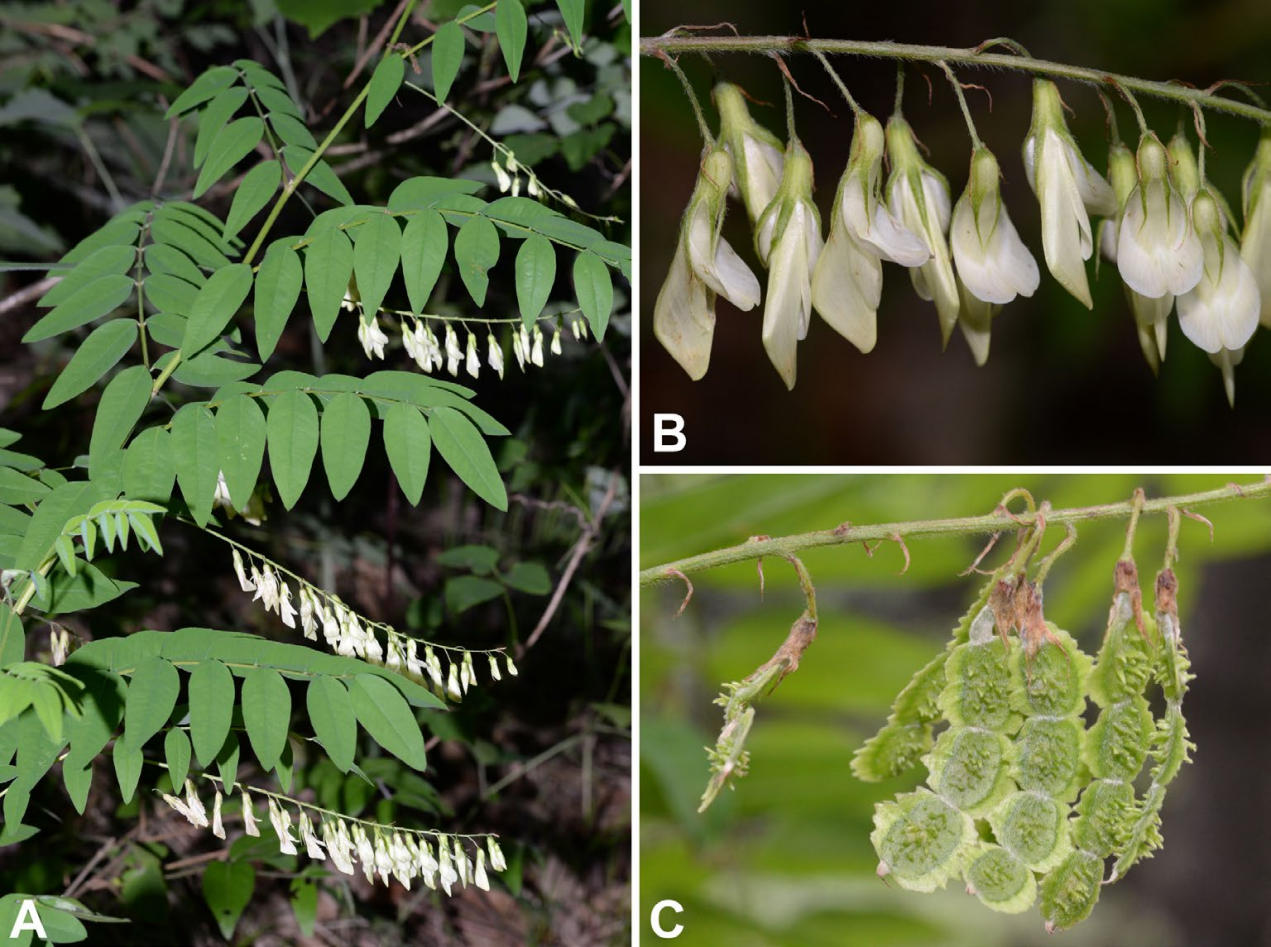
Photographs of *Hedysarum dentatoalatum* from the field. (A) Upper part of a flowering plant; (B) flowers; (C) legumes. Photoed by Pei‐Liang Liu.

**Description**. Perennial herbaceous plants, 0.6–1.0 m tall; main root 8–12 mm in diameter, woody. Stems cespitose, erect or ascending, upper part branched; internodes densely pubescent. Leaves imparipinnate, alternate, 11–25 cm long; stipules connate, opposite to leaves, membranous, brown, pubescent, lower ones 10–15 mm long, becoming smaller in upper part of stem; rachises sparsely or densely pubescent; leaflets 11–23, opposite or alternate; petiolules 1.8–2.2 mm long, pubescent; leaflet blades ovate or ovate‐lanceolate, 35–50 × 10–22 mm, membranaceous, glabrous adaxially, sparsely pubescent abaxially, base broadly cuneate, apex acute and aristulate. Racemes axillary, including peduncles 12–22 cm long, with 15–35 flowers, peduncles sparsely pubescent; pedicels 7–10 mm long, sparsely pubescent; bracts linear, pubescent, 4–6 mm long; bracteoles 2, linear, 2–6 mm long; calyx tube 3–4 mm long, pubescent; calyx teeth 5, pubescent, linear‐triangular, the two adaxial teeth 4–6 mm long, the two lateral teeth 4–7 mm long, the abaxial tooth 4–8 mm long; corolla pale yellow; standard petal obovate, 12–14 × 5–7 mm, apex retuse, base attenuate; wing petals 13–15 × 2.0–2.5 mm, auricle linear, as long as claw, ca. 3 mm long; keel petals 17–19 × 6–7 mm, auricle triangular, ca. 0.5 mm long; androecium diadelphous, 18–19 mm long; ovary linear, glabrous, style 14–15 mm long. Legume a loment, divided into 2–5 articles; articles suborbicular or oblong, compressed, 8–12 × 10–12 mm, pubescent, with reticulate veins, with prickles on the veins, with wings and prominent teeth along both sutures.

**Phenology**. Flowering in May and June; fruiting in June; seeds mature in September.

**Distribution and Habitat**. *Hedysarum dentatoalatum* is distributed in southeastern Shaanxi and western Henan in China (Figure 3). It grows in temperate mixed coniferous‐broadleaf forest (dominated by *Q. aliena* var. *acuteserrata*, *Q. acutissima* Carruth. and *P. tabuliformis* ), 1200–1500 m a.s.l.

**Specimens Examined**. CHINA, Shaanxi Province, Shangxian County (now Shangzhou District), Niutouya, on slope in forest, 1200 m a.s.l., 22 June 1952, *T.P.Wang 15466* (Holotype, WUK!; Isotypes, PE!, KUN!); Shanyang County, Wangzhuang, 1460 m a.s.l., 6 May 1985, *W.N.Wang S006* (WNU!); Shanyang County, 33°41′18.6″ N, 110°0′3.7″ E, 1440 m a.s.l., 17 May 2021, *P.L.Liu 1107* (WNU! & WUK!). Henan Province, Luanchuan County, 1400 m a.s.l., 21 June 2013, *Z.Y.Chang* et al. *2013267* (WUK!); Songxian County, Baihe, 1500 m a.s.l., 14 September 1963, *Anonym 5193* (HEAC!).

## 6 Conclusion

This paper describes *H. langranii* as a new species based on morphological and phylogenetic evidence. The new species is morphologically similar to *H. smithianum*, but they are allopatric in distribution. The new species is substantially different from *H. dentatoalatum* in morphology, and these two species exhibit altitudinal segregation. The incongruent phylogenetic position of *H. dentatoalatum* in the nuclear and plastid trees requires further investigation to determine the reason for this incongruence. *Hedysarum langranii* and its relatives represent a good system for investigating plant speciation.

## Author Contributions

**Pei‐Liang Liu:** conceptualization (lead), data curation (lead), formal analysis (lead), funding acquisition (equal), investigation (lead), methodology (lead), visualization (lead), writing – original draft (lead). **Yong‐Xing Qin:** data curation (supporting), investigation (supporting), methodology (supporting), visualization (supporting), writing – original draft (supporting). **Lu‐Lu Xun:** resources (equal), validation (equal), writing – review and editing (equal). **Bin Li:** funding acquisition (equal), project administration (equal), resources (equal), validation (equal), writing – review and editing (equal). **Yuan Lu:** resources (equal), validation (equal), writing – review and editing (equal). **Ming Yue:** conceptualization (supporting), funding acquisition (equal), project administration (equal), resources (equal), supervision (lead), writing – review and editing (equal).

## Funding

This research was funded by the National Natural Science Foundation of China (grant number 31900179) and the Basic Research Program of the Shaanxi Academy of Sciences (grant number 2023K‐14).

## Conflicts of Interest

The authors declare no conflicts of interest.

## Data Availability

The newly generated DNA sequences have been deposited in GenBank and the accession numbers are available in Appendix 1.

## Appendix 1

Taxon name, geographical locality, voucher, herbarium code, and GenBank accession number for the sequences used in this study. For each sample, accession numbers are given for the ETS, ITS, *psb*A‐*trn*H, *trn*C‐*pet*N, *trn*L‐F, *trn*S‐G, and *pet*N‐*psb*M sequences. A dash (—) indicates a missing sequence. New sequences generated in this study are indicated by an asterisk (*).

*Hedysarum algidum* L.Z.Shue: China, Gansu, *W.Q.Yang 2008010* (WUK), KY365837, KP338149, KP338400, KY366037, KP338272, KY365888, KY365987;

*Hedysarum alpinum* L.: Russia, Buryatia, *I.Yu. Selyutina N.A. BBB_18082017* (AIMAP), —, MT081327, —, —, —, —, —;

*Hedysarum alpinum* L.: Russia, Buryatia, *S.G.Kazanovsky & U.N.Pochinnik NSK0013946* (NSK), —, —, —, —, MG905923, —, —;

*Hedysarum americanum* (Michx.) Britton: Canada, Tuktut Nogait National Park, *L.J.Gillespie* et al. *8934* (US), KY365838, KP338150, KP338402, KY366038, KY366134, KY365889, KY365988;

*Hedysarum astragaloides* Benth. ex Baker: Pakistan, Punjab, *W.Koelz 5024* (US), KY367282, KP338153, KP338405, KY367325, KP338275, OR971769, KY367309;

*Hedysarum cachemirianum* Benth. ex Baker: Kashmir, Kishenganga Valley and the road to Nanga Parbat, *R.R. & I.D.Stewart 18354* (US), KY367283, KY367300, KY367343, KY367326, OR971763, —, KY367310;

*Hedysarum campylocarpon* H.Ohashi: China, Xizang, Jilong, *Z.Y.Chang* et al. *2013073* (WUK), KY367284, KY367301, KY367344, KY367327, —, —, KY367311;

*Hedysarum campylocarpon* H.Ohashi: China, Xizang, Nielamu, *Z.Y.Chang* et al. *2011203* (WUK), —, —, —, —, KP338279, OR971765, —;

*Hedysarum chinense* (B.Fedtsch.) Hand.‐Mazz.: China, Sichuan, Pingwu, *L.M.Lu 2008343* (PE), PX610178*, PX612368*, PX610190*, PX610202*, PX610214*, PX610226*, —;

*Hedysarum chinense* (B.Fedtsch.) Hand.‐Mazz.: China, Sichuan, Maowen, *Chuanjing'a (59) 5172* (WUK), PX610175*, PX612365*, —, —, —, —, —;

*Hedysarum chinense* (B.Fedtsch.) Hand.‐Mazz.: China, Sichuan, Mianning, *Anonym 0183* (SM), PX610176*, PX612366*, —, —, PX610213*, —, —;

*Hedysarum chinense* (B.Fedtsch.) Hand.‐Mazz.: China, Sichuan, Mianzhu, *Mianzhudui 520* (SM), PX610177*, PX612367*, —, —, —, —, —;

*Hedysarum cisdarvasicum* Kamelin & Karimova: Tajikistan, Darvaz, Yakhsu river basin, *R.Kamelin s.n*. (TAD), MK639303, MK639233, MK639289, — MK639275, MK639261, MK639247;

*Hedysarum citrinum* E.G.Baker: China, Xizang, Longzi, *Y.S.Chen* et al. *13‐468* (WUK), OR971731, OR982385, OR971741, OR971748, OR971757, OR971778, —;

*Hedysarum cuonanum* P.L.Liu, J.Wen & Zhao Y.Chang: China, Xizang, Cuona, *Y.S.Chen* et al. *13‐0948* (WUK), KY367286, KY367302, KY367345, KY367329, OR971764, OR971767, KY367312;

*Hedysarum dentatoalatum* K.T.Fu: China, Shaanxi, Shanyang, *P.L.Liu 1107‐1* (WNU), PX610179*, PX612369*, PX610191*, PX610203*, PX610215*, PX610227*, PX610238*;

*Hedysarum dentatoalatum* K.T.Fu: China, Shaanxi, Shanyang, *P.L.Liu 1107‐5* (WNU), PX610180*, PX612370*, PX610192*, PX610204*, PX610216*, PX610228*, PX610239*;

*Hedysarum dentatoalatum* K.T.Fu: China, Henna, Luanchuan, *Z.Y.Chang* et al. *2013267* (WUK), KY365844, KP338162, KP338415, KY366044, KP338283, KY365893, KY365994;

*Hedysarum denticulatum* Regel: Kyrgyzstan, Osh, Alay Valley, *I.Sodombekov & N.Rogova KPL_00816* (MO), KY365845, KY366156, KY365760, —, KY366137, KY365894, KY365995;

*Hedysarum falconeri* Baker, Pakistan, Karakoram, *O.Polunin 6096* (F), KY367287, KP338163, KP338416, KY367330, KP338284, OR971768, KY367313;

*Hedysarum hedysaroides* Schinz & Thell.: Russia, Kamchatka, Tolbachik Volcano, *S.McDonald & N.A.Brummitt 23* (US), KY365847, KP338168, KP338421, KY366046, KP338288, KY365896, KY365997;

*Hedysarum inundatum* Turcz.: China, Shanxi, *Huangtudui 01313* (WUK), KY365848, KP338170, KP338423, KY366047, KP338290, KY365897, KY365998;

*Hedysarum kumaonense* Benth. ex Baker: China, Xizang, Jilong, *Z.Y.Chang* et al. *2013084* (WUK), KY367288, KP338174, KP338427, KY367331, KP338294, KY365899, KY367314;

*Hedysarum langranii* P.L.Liu: China, Shaanxi, Zhashui, *Z.Y.Chang* et al. *2013269* (WUK), PX610187*, PX612377*, PX610199*, PX610211*, PX610223*, PX610235*, —;

*Hedysarum langranii* P.L.Liu: China, Shaanxi, Baoji, *R.B.Zhu 20130925* (WUK), PX610181*, PX612371*, PX610193*, PX610205*, PX610217*, PX610229*, PX610240*;

*Hedysarum langranii* P.L.Liu: China, Henan, Luanchuan, *P.L.Liu 576* (WNU), PX610185*, PX612375*, PX610197*, PX610209*, PX610221*, PX610233*, PX610244*;

*Hedysarum langranii* P.L.Liu: China, Henan, Huixian, *P.L.Liu 1157‐1* (WNU), PX610183*, PX612373*, PX610195*, PX610207*, PX610219*, PX610231*, PX610242*;

*Hedysarum langranii* P.L.Liu: China, Henan, Huixian, *P.L.Liu 1157‐6* (WNU), PX610184*, PX612374*, PX610196*, PX610208*, PX610220*, PX610232*, PX610243*;

*Hedysarum langranii* P.L.Liu: China, Shaanxi, Chang'an, *Z.Y.Chang* et al. *2016151‐1* (WUK), PX610182*, PX612372*, PX610194*, PX610206*, PX610218*, PX610230*, PX610241*;

*Hedysarum langranii* P.L.Liu: China, Shaanxi, Luonan, *P.L.Liu 1647* (WNU), PX610186*, PX612376*, PX610198*, PX610210*, PX610222*, PX610234*, PX610245*;

*Hedysarum langranii* P.L.Liu: China, Shaanxi, Ningshan, *Z.M.Jiang 1565* (WUK), KY365842, KP338159, KP338412, KY366042, KP338280, KY365891, KY365992;

*Hedysarum lehmannianum* Bunge: Tajikistan, Zeravshan range, Pastrud‐darya river basin, Turzun, Abdusalyamova, *Zhogoleva & Ovchinnikov 4932* (TAD), MK639308, MK639238, MK639294, —, MK639280, MK639266, MK639252;

*Hedysarum longigynophorum* C.C.Ni: China, Xizang, Gongbujiangda, *Z.Y.Chang* et al. *QZ620* (WUK), KY367290, KP338175, KP338428, KY367333, KP338295, KY365900, KY367316;

*Hedysarum minjanense* Rech.f.: China, Xinjiang, Tashikuergan, *Xizhixinjiangdui 1091* (WUK), KY365852, KY366159, KY365763, —, KY366140, KY365902, KY366001;

*Hedysarum nagarzense* C.C.Ni: China, Xizang, Langkazi, *L.R.Xu 1463* (WUK), KY367292, KY367305, KY367348, KY367335, OR971762, OR971770, KY367318;

*Hedysarum neglectum* Ledeb.: China, Xinjiang, Xinyuan, *L.R.Xu 1533* (WUK), KY365853, KY366160, KY365764, KY366050, KY366141, KY365903, KY366002;

*Hedysarum polybotrys* Hand.‐Mazz.: China, Gansu, Xiahe, *Z.Y.Chang* et al. *QZ042* (WUK), KY365854, KP338182, KP338434, KY366051, KP338300, KY365905, KY366003;

*Hedysarum przewalskii* Yakovlev: China, Ningxia, Helanshan, *R.B.Zhu s.n*. (WUK), OR971730, OR982389, OR971740, OR971750, OR971760, OR971776, —;

*Hedysarum qilianshanense* P.L.Liu: China, Gansu, Su'nan, Xiaogushan, *P.L.Liu 458* (WUK), OR971726, OR982388, OR971739, OR971745, OR971756, OR971773, OR971734;

*Hedysarum semenowii* Regel & Herder: Kazakhstan, Ulken, *I.Roldugin 4823* (US), KY365856, KP338183, KP338435, KY366053, KP338301, KY365907, KY366005;

*Hedysarum sikkimense* Benth. ex Baker: China, Xizang, Yadong *Y.S.Chen* et al. *13‐1772* (WUK), KY367294, KY367306, KY367349, KY367337, OR971753, OR971771, KY367320;

*Hedysarum smithianum* Hand.‐Mazz.: China, Shanxi, Huoxian, *T.W.Liu s.n*. (PE), PX610188*, PX612378*, PX610200*, —, PX610224*, PX610236*, —;

*Hedysarum smithianum* Hand.‐Mazz.: China, Shanxi, Qinxian, *K.J.Guan & Y.L.Chen 1096* (WUK), PX610189*, PX612379*, PX610201*, PX610212*, PX610225*, PX610237*, —;

*Hedysarum taipeicum* (Hand.‐Mazz.) K.T.Fu: China, Shaanxi, Mt. Taibai, *P.L.Liu 681‐1* (WNU), OR971727, OR982391, OR971742, OR971752, OR971759, OR971777, OR971736;

*Hedysarum tanguticum* B.Fedtsch.: China, Qinghai, Chengduo, *Z.Y.Chang* et al. *2010230* (WUK), KY365857, KP338188, KP338440, KY366056, KP338306, KY365910, KY366008;

*Hedysarum tibeticum* (Benth.) B.H.Choi & H.Ohashi: China, Xizang, Langkazi, *Z.Y.Chang* et al. *2011111* (WUK), KY367296, KP338189, KP338441, KY367339, KP338307, KY365911, KY367321;

*Hedysarum ussuriense* I.Schischkin & Kom.: China, Jilin, Mt. Changbai, *M.Z.Sun s.n*. (WNU), OR971728, OR982384, OR971743, OR971749, OR971758, —, OR971735;

*Hedysarum wangii* P.L.Liu & Zhao Y.Chang: China, Gansu, Xiahe, Qu'ao, *P.L.Liu 432* (WUK and WNU), MK639312, MK639242, MK639298, OR971744, MK639284, MK639270, MK639256;

*Hedysarum xizangense* C.C.Ni: China, Xizang, Longzi, Sananqulin, *Y.S.Chen* et al. *13‐0886* (WUK), KY367297, KY367307, KY367350, KY367340, KP338310, OR971766, KY367322.
